# The Relationship between Hepatic Myeloid-Derived Suppressor Cells and Clinicopathological Parameters in Patients with Chronic Liver Disease

**DOI:** 10.1155/2021/6612477

**Published:** 2021-03-29

**Authors:** Zhijia Zhou, Penghua Lai, Shaoliang Zhang, Yujie Wang, Ning Qu, Dawei Lu, Liangqin Gao, Lingxia Xu, Yanmiao Yang, Ting Zhang, Xue Sun, Xiaoting Zheng, Yaoyu Liu, Huiqing Liang, Shaodong Chen

**Affiliations:** ^1^Department of Traditional Chinese Medicine, School of Medicine, Xiamen University, Xiamen, Fujian, China; ^2^Hospital of Xiamen University, Xiamen, Fujian, China; ^3^Fujian University of Traditional Chinese Medicine, Fujian, China; ^4^Liver Disease Center, Xiamen Hospital of Traditional Chinese Medicine, Xiamen, China

## Abstract

Myeloid-derived suppressor cells (MDSCs) have attracted attention due to their important role in inflammation. Several studies have investigated the involvement of MDSCs in chronic liver disease. However, due to the difference of MDSC phenotypes, patient types, and sample sources among the studies, the results are inconsistent and controversial. We took advantage of a large well-defined cohort of 98 (24 patients with CHB, 18 with NAFLD, 13 with HCC, 16 with PBC, and 27 with AIH) patients with liver inflammation and 12 healthy controls to investigate the expression of MDSCs, and the relationships between the expression of hepatic MDSCs and the clinical characteristics were analyzed. We found that the expression of CD11b+CD33+ MDSCs is closely related to chronic liver disease and positively correlated with clinical parameters such as ALT, AST, and globulin. Ultimately, the present study suggests that hepatic CD11b+CD33+ MDSCs are increased in HCC and AIH and positively correlate with the liver stages of hepatitis activity and liver fibrosis stage.

## 1. Introduction

Chronic liver disease (CLD) refers to long-term liver fibrosis due to various causes, which eventually leads to liver cirrhosis and leads to a poor prognosis. The threat of liver disease is increasing due to the combined effects of alcohol-related liver disease (ARLD) [1], nonalcoholic fatty liver disease (NAFLD) [2], and viral hepatitis [3]. It has been reported that disorders of the immune system could contribute to inflammation and cirrhosis, including intrahepatocyte reactions, natural killer cells (NK cells), natural killer T cells (NK-T cells), and monocytes [4, 5].

There are complex and heterogeneous regulatory cell populations that may regulate inflammation in the liver-specific immune response, including myeloid-derived suppressor cells derived from bone marrow [6]. The special mechanism of MDSCs inhibiting host immune response under pathological conditions has attracted the interest of researchers in various fields. Numerous studies have been carried out to define biomarkers for the identification of different subpopulations of MDSCs in various pathological conditions. Human MDSCs are phenotypically characterized as CD11b+, CD33+, and HLA-DR- and can be divided into granulocytic (CD14-/CD15+/CD66b+) and monocytic (CD14+) subtypes.

The accumulation of MDSCs is a complex and gradual phenomenon governed by multiple factors. In response to chronic infection and inflammation, signals including GM-CSF, G-CSF, M-CSF, S-SCF, VEGF, and polyunsaturated fatty acids are produced to expand the immature myeloid cells [7, 8]. Furthermore, chronic liver disease caused by different pathogenic factors, such as steatosis, virus, autoimmune inflammation, and tumor, could influence the local microenvironment, including cytokines, vascular growth factors, and the influence of the major histocompatibility complex [9, 10]. Some studies have shown a significant correlation between MDSCs and clinical cancer stage such as circulating MDSCs in breast cancer patients [11]. However, correlations between MDSCs levels and clinical parameters are contradictory, but this could be due to the heterogeneous nature of human MDSCs and lack of uniform markers for accurate characterization of human MDSCs [12].

Only limited data is available on the involvement of MDSC in chronic liver injury and the development of liver fibrosis. And most of the studies consider only circulating MDSC, but some attention should also be paid to tumor-infiltrating cells. Until recently, histological studies have presented technical challenges since multiple markers are required to identify MDSCs. The introduction of multiplex immunohistochemistry and new markers of MDSC could help address this problem.

In our study, we investigated the relationship between the expression of MDSCs in patients of liver disease and clinical parameters by IF and IHC methods.

## 2. Materials and Methods

### 2.1. Clinical Data

During the period between September 2018 and June 2019, 98 patients with chronic liver disease and 12 healthy controls (HC) were enrolled in this study who all underwent percutaneous liver biopsy in Xiamen Hospital of Traditional Chinese Medicine. In 24 patients with chronic hepatitis B (CHB), CHB diagnosis was made according to the European Association for the Study of the Liver (EASL) clinical practice guidelines [13]. In 18 with nonalcoholic fatty liver disease (NAFLD), guidelines for the diagnosis of NAFLD proposed by the American Association for the Study of Liver Diseases were used [14]. In 13 with hepatocellular carcinoma (HCC), all enrolled patients with HCC met the diagnostic criteria of the American Association for the Study of Liver Diseases [15]. In 16 subjects with primary biliary cirrhosis (PBC), the diagnostic criteria for PBC were based on the guidelines for the diagnosis and treatment of PBC prepared by the American Association for the Study of Liver Diseases [16]. In 27 with autoimmune hepatitis (AIH), all the AIH patients were native Chinese living in China and satisfied the criteria of the International Autoimmune Hepatitis Group for diagnosis of AIH [17]. Age- and gender-matched healthy controls (*n* = 20) consisted of local volunteers. HC inclusion criteria were as follows: no history of chronic diseases, no acute and chronic infection caused by bacteria, fungi, viruses, and pathogens, no pregnancy, and normal serum biochemistry (Table 1). The following data were collected at the time of enrolment of patients: age, gender, body mass index, alanine aminotransferase, aspartate aminotransferase, albumin, gamma-glutamyl transferase, cholesterol, triglyceride, fasting plasma glucose, uric acid, total bilirubin, direct bilirubin, alpha fetoprotein, white blood cells, neutrophils, lymphocytes, platelet, stages of hepatitis activity, and liver fibrosis stage. Particularly, the Scheuer scoring system was used to assess the stages of hepatitis activity and liver fibrosis stage from G0 to G4 and S0 to S4. According to the Scheuer scoring system, the hepatic inflammation activity grade (G) was divided into G0 (no hepatic necroinflammation) and G1 (inflammation but no necrosis), G2 (focal necrosis or acidophil bodies), G3 (severe focal cell damage), and G4 (damage including bridging necrosis), and the liver fibrosis stage (S) was divided into S0 (no fibrosis), S1 (enlarged fibrotic portal tracts), S2 (periportal or portal-portal septa but intact architecture), S3 (fibrosis with architectural distortion but no obvious cirrhosis), and S4 (probable or definite cirrhosis). This study was approved by the Clinical Ethics Review Board of the Xiamen Hospital of Traditional Chinese Medicine.

### 2.2. Immunohistochemistry and Immunofluorescence Double Staining

All liver samples were fixed in 10% neutral buffered formalin and embedded in paraffin, and 4 *μ*m thick sections were cut from each paraffin block. Immunohistochemistry was performed on the liver sections with antibodies against CD11b (1 : 100 dilution; Abcam, Cambridge, United Kingdom). All sections were visualized by light microscopy, and five fields were randomly selected for analysis; the numbers of CD11b-positive cells were quantified at 40 × 10 magnification, and the expression degrees of CD11b were scored by an unbiased observer. For double immunofluorescence of liver samples, sections of liver tissues were incubated with CD11b (1 : 100 dilution; Abcam, Cambridge, United Kingdom) and CD33 (1 : 100 dilution; Cambridge, United Kingdom) separately for 30 min at 37°C. The nucleus was stained with DAPI (Cell Signaling Technology, Boston, USA). Confocal scanning was performed using a laser scanning confocal microscope (LSM-710, Carl Zeiss, Jena, Germany). The cell positivity of both markers (“merge”) was assessed through the program ImageJ software (ImageJ 1.43u, NIMH, Bethesda, Maryland, USA, https://imagej.nih.gov/ij/). The examination and the computer analysis of the histological sections were performed without knowledge of the origin of the tissue samples.

### 2.3. Statistical Analyses

The collected data were analyzed statistically with SPSS 20.0, and Prism 8 software (GraphPad Software) was used to create the graphs and implement the statistical analysis. Post hoc pairwise comparison was carried out using the least significant difference *t*-test, and comparison of data at multiple time points was carried out using the variance of repeated measures and expressed as *F*. The posttest was performed using Bonferroni. The relationships of clinical parameters were evaluated using R (https://www.r-project.org/) (version 3.6, cutoff = 0.05). For all tests, a *p* value < 0.05 was considered statistically significant.

## 3. Results

### 3.1. CD11b Upregulated in the Liver of Liver Disease Patients

**Figure 1:**
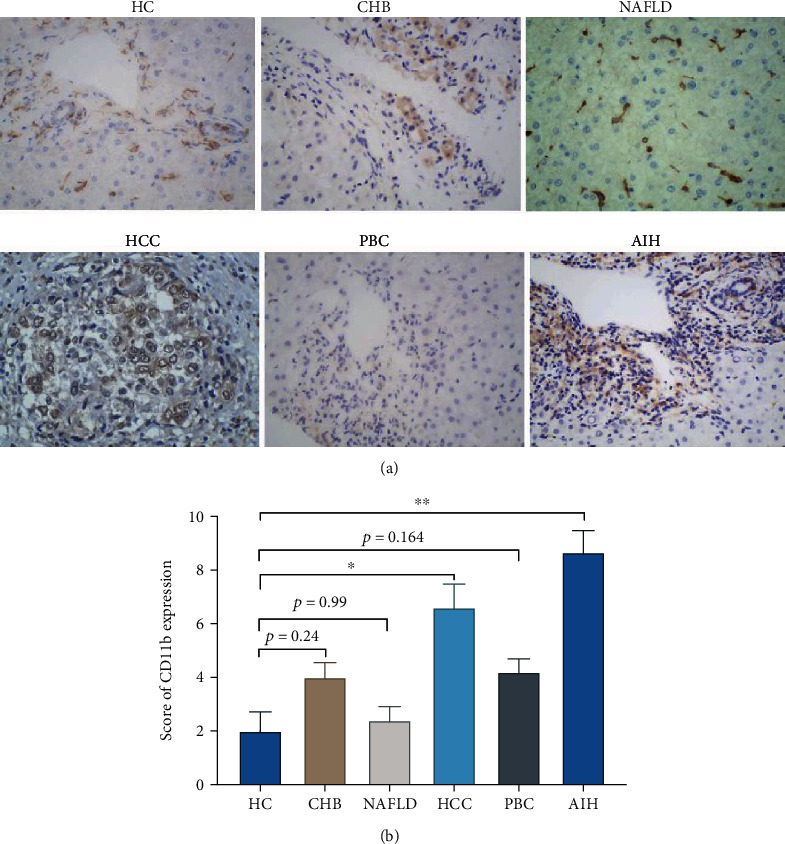
Immunohistochemistry analysis of the expression of CD11b in the liver: (a) representative staining images and (b) statistical analysis of CD11b in HC, CHB, NAFLD, HCC, PBC, and AIH. ^∗^*p* < 0.05, ^∗∗^*p* < 0.01, and ^∗∗∗^*p* < 0.001.

To confirm that CD11b plays an important role in the pathogenesis of CLD, we investigated the expression of CD11b in the liver by immunohistochemistry.  Figure 1(a) shows a semiquantitative method to determine the protein expression level, with 0-4 being low expression and 5-12 being high expression. CD11b is lowly expressed in HC, NAFLD, CHB, and PBC and highly expressed in HCC and AIH. Immunohistochemical results also showed that the expression of CD11b in autoimmune liver disease (AIH) tissues was significantly higher than that in normal tissues (*p* < 0.01). In addition, patients with HCC showed significantly increased levels of CD11b compared to HCs (*p* < 0.05), but there was no significant difference in CHB (*p* = 0.24), NAFLD (*p* = 0.99), and PBC (*p* = 0.164), compared to HC (Figure 1(b)).

### 3.2. The Expression of Hepatic CD11b+CD33+ Cells Is Connected to the Development of Chronic Liver Disease

**Figure 2:**
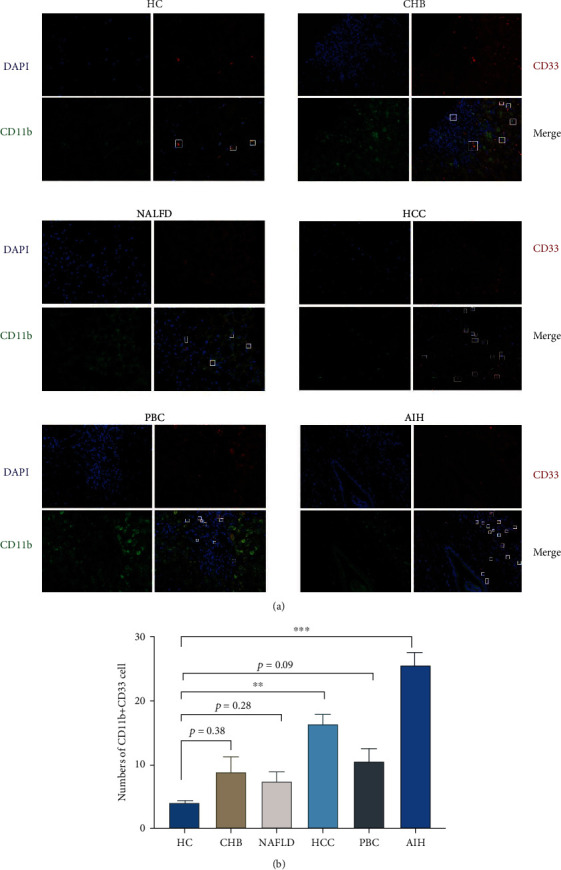
CD11b+CD33+ cells in chronic diverse disease by immunofluorescence double staining. Representative confocal staining (a) and analysis (b) of CD11b (in green), CD33 (in red), and DAPI (for nuclei in blue) in the livers of HC, NAFLD, LC, AIH, PBC, CHB, and HCC. DAPI: 40,6-diamidino-2-phenylindole. ^∗^*p* < 0.05, ^∗∗^*p* < 0.01, and ^∗∗∗^*p* < 0.001.

To study the expression of hepatic CD11b+CD33+ cells in different liver diseases, immunofluorescence is used to represent the percentages of hepatic CD11b+CD33+ cells. Confocal staining demonstrated that CD33 and CD11b were colocalized in the AIH liver (Figure 2(a)). As shown in Figure 2, the percentages of MDSCs were significantly higher in the AIH (*p* < 0.001) and HCC (*p* < 0.01) patients compared to healthy donors. Importantly, the expression of hepatic CD11b+CD33+ cells was the highest in AIH patients. As we all know, the lack of immune response to specific antigens is one of the main causes of autoimmune diseases, and it was reported that MDSCs are related to immune suppression [18, 19]. However, compared with healthy donors, there was no significant difference in CHB (*p* = 0.28), NAFLD (*p* = 0.38), and PBC (*p* = 0.09) (Figure 2(b)).

### 3.3. Correlation between Hepatic CD11b+CD33+ Cells and Biochemical Parameters in Patients with CLD

**Figure 3:**
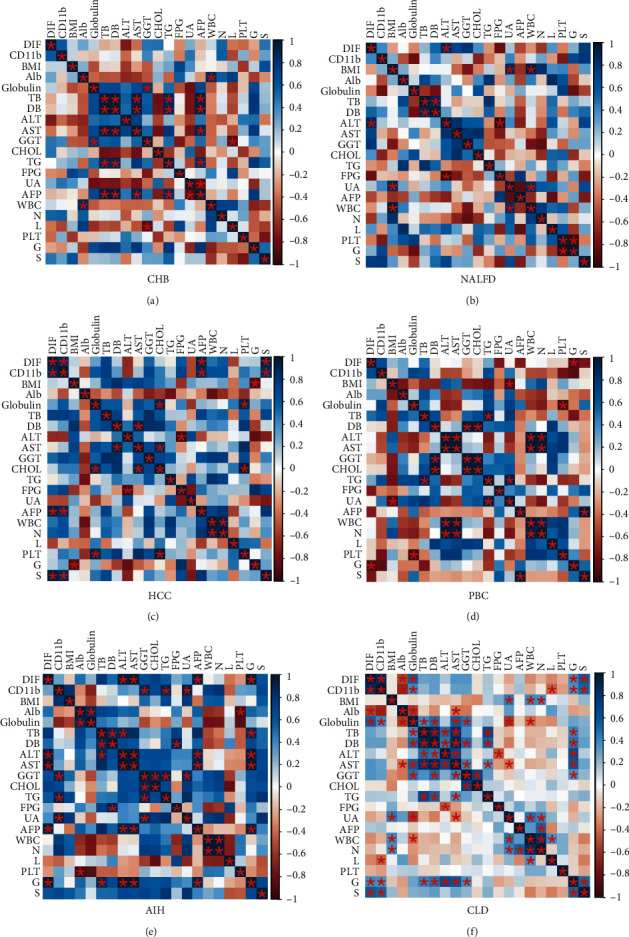
Correlation analysis of CD11b+CD33+ expression and CD11b with clinical parameters in patients with CHB (a), NAFLD (b), HCC (c), PBC (d), AIH (e), and CLD (f). ^∗^*p* < 0.05. DIF: double immunofluorescence of CD11b and CD33; BMI: body mass index; ALT: alanine aminotransferase; AST: aspartate aminotransferase; Alb: albumin; GGT: gamma-glutamyl transferase; CHOL: cholesterol; TG: triglyceride; FPG: fasting plasma glucose; UA: uric acid; TB: total bilirubin; DB: direct bilirubin; AFP: alpha fetoprotein; WBC: white blood cells; N: neutrophils; L: lymphocytes; PLT: platelet; G: stages of hepatitis activity; S: liver fibrosis stage.

To assess the potential role of hepatic CD11b+CD33+ cells (double immunofluorescence (DIF) of CD11b and CD33) in CLD patients, we analyzed the differences in physical and chemical parameters of different disease states in chronic liver disease. Multivariate analysis of chemical parameters using the R package Corrplot revealed that, for patients with CHB, serum AST levels were positively correlated with TB (*p* < 0.05) and DB (*p* < 0.05). Moreover, the parameters with positive correlation were as follows: DB and TB (*p* < 0.05), GLb and GGT (*p* < 0.05), WBC and Alb (*p* < 0.05), TG and DB (*p* < 0.05), TG and DB (*p* < 0.05), and AFP and TB, DB, AST, and TG (*p* < 0.05), whereas it was negatively correlated with L and GGT (*p* < 0.05) (Figure 3(a)). However, there were no significant correlations between DIF (CD33+CD11b+ expression) and clinic parameters. As was shown in Table 1 and Figure 3(b), there were positive correlations between DIF and ALT (*p* < 0.05), UA and BMI (*p* < 0.05), and WBC and BMI (*p* < 0.05) in patients with NAFLD. In addition, a negative correlation was found between FPG and ALT (*p* < 0.05). Physiological parameters were further investigated with patients with HCC, and the parameters with positive correlation are as follows: DIF and CD11b (*p* < 0.05), DIF and AFP (*p* < 0.05), DIF and S (*p* < 0.05), CD11b and AFP (*p* < 0.05), CD11b and S (*p* < 0.05), CHOL and Glb (*p* < 0.05), CHOL and AST (*p* < 0.05), AST and DB (*p* < 0.05), PLT and Glb (*p* < 0.05), and PLT and CHOL (*p* < 0.05). Furthermore, the levels of BMI and G (*p* < 0.05) and FPG and ALT (*p* < 0.05) were significantly negatively correlated in patients with HCC (Figure 3(c)). For patients with PBC, DIF was inversely correlated with G (*p* < 0.05) and UA was negatively correlated with BMI (*p* < 0.05). In contrast, GGT (*p* < 0.05) and CHOL (*p* < 0.05) were positively correlated with DB. ALT and AST also were positively correlated with WBC and N (Figure 3(d)). For patients with AIH, we revealed that there were positive correlations between DIF and ALT (*p* < 0.05), ALT (*p* < 0.05), and AFP (*p* < 0.05). The expression of CD11b was positively correlated with several clinic parameters including GGT (*p* < 0.05), TG (*p* < 0.05), and UA (*p* < 0.05). Of note, stages of hepatitis activity (G) were positively correlated with DIF, AST, ALT, AFP, and TB (*p* < 0.05). In addition, the parameters with positive correlation are as follows: Alb and Glb (*p* < 0.05), DB and TB (*p* < 0.05), DB and FPG (*p* < 0.05), ALT and AFP (*p* < 0.05), AST and AFP (*p* < 0.05), GGT and TG (*p* < 0.05), and GGT and CHOL, UA, G, TG, and G (*p* < 0.05). However, PLT was negatively correlated with Glb (*p* < 0.05) (Figure 3(e)). As shown in Figure 3(f), a total of 39 groups were found to be statistically correlated with at least one of the physiological parameters (*p* < 0.05). Importantly, there was statistical support for correlations between CD33+CD11b+ cells and clinic parameters. The expression of CD11b, Glb, G, and S was positively correlated with DIF, and Alb was negatively correlated with DIF. Moreover, the levels of Glb, S, and G were positively correlated with the expression of CD11b. However, Alb was also negatively correlated with the expression of CD11b. In addition, the stages of hepatitis activity (G) played a vital role in the development of chronic liver disease. G was positively correlated with several clinic parameters including TB, DB, ALT, AST, and GGT (Figure 3(f)).

## 4. Discussion

MDSCs are necessary for regulating immune responses during autoimmune diseases. As one of the important components of the immunosuppressive network, MDSCs have been shown to facilitate tumor formation by blocking the host immune system [6]. Recent studies support the role of MDSCs as essential regulators of chronic inflammatory liver diseases, and MDSCs are expanded in pathological conditions such as malignancy or infection and suppress antitumor immunity [10], and the liver is an important site of MDSC induction for extrahepatic infections and cancer [20–22]. The expression of CD33 and CD11b on activated monocytes and macrophages was associated with the disease progression and poor survival of patients [23]. However, the expression and significance of CD11b+CD33+ MDSCs in CLD patients remain to be elucidated.

In our current study, we have elaborated that the expression of CD11b in the liver of AIH and HCC patients was significantly increased compared with that of healthy control. Of note, we have observed that there were significant differences in the expression level of hepatic CD11b+CD33+ MDSCs in AIH and HCC during liver inflammation. Furthermore, we reported the correlation between the levels of hepatic CD11b+CD33+ MDSCs and clinic biochemical parameters in CLD. In addition, we demonstrated that some parameters in the clinic have intriguing correlations during the development of CLD. Ultimately, we found that the expression of hepatic CD11b+CD33+ MDSCs was increased in chronic liver diseases such as autoimmune hepatitis and hepatocellular carcinoma and positively correlated with the liver stages of hepatitis activity or liver fibrosis stage.

As precursors of macrophages, granulocytes, and dendritic cells, MDSCs represent a heterogeneous population of bone marrow-derived myeloid progenitors that fail to differentiate into mature myeloid cells and expand under pathological conditions until they are detectable in the blood, peripheral lymphoid tissues, cancer tissues, and inflamed sites. Murine MDSCs are identified as coexpressing Gr-1 and CD11b markers, and human MDSCs are characterized by the expression of CD33. Previous research has shown that CD11b+CD33+ MDSCs play a vital role in the development of AMT [24, 25] and inflammation [26].

In our research, we have figured that hepatic CD11b+CD33+ MDSCs are significantly elevated in patients with AIH and HCC. Although this finding is not altogether surprising as MDSC levels are often increased in patients with cancer, we have formally shown that MDSC accumulation occurs concurrently with the expression of ITGAM in HCC. ITGAM, also known as CD11b, was identified as associated with systemic lupus erythematosus [27] and multiple myeloma [28]. We observed that the expression of CD11b was significantly elevated in AIH and HCC compared to healthy controls. Of note, the expression of CD11b was positively correlated with several clinic parameters including GGT, TG, and UA in patients with AIH. As we all know, GGT is positively correlated with the severity of liver disease. Palle et al. had demonstrated that more AIH in African Americans presented with end-stage liver disease (ESLD) with high GGT [29]. Recently, lipid metabolism has been found to play an important role in the developmental differentiation and typing of various immune cells. Recent research showed that hepatic triglycerides and hepatic CD11b+ macrophages were increased in NALFD 19-month-old aged female mice [30]. Moreover, it has been found that UA levels are elevated in immune system diseases with positive autoimmune antibodies, such as Sjogren's syndrome, Hashimoto's thyroiditis, systemic lupus erythematosus, and systemic sclerosis [31, 32]. It is well known that the immune system is closely related to the endocrine system. Those findings suggest that TG and UA have an important impact on the immune system. However, the underlying mechanism of the correlation between the expression of CD11b and GGT in patients with AIH needs to be further clarified.

Another important finding of our study is that we examined the expression of hepatic CD11b+CD33+ MDSCs in liver biopsy of 24 patients with CHB, 18 with NAFLD, 13 with HCC, 16 with PBC, and 27 with AIH by immunofluorescence analysis. The expression of hepatic CD11b+CD33+ MDSCs in AIH and HCC patients was significantly increased compared to that in healthy controls. It was positively correlated with the degree of liver fibrosis and inflammation in patients with AIH and PBC. As is well known, liver fibrosis and inflammation are some of the key pathological features of chronic liver diseases [33]. This finding suggests that hepatic CD11b+CD33+ MDSCs may be involved in the progression of chronic liver disease.

In particular, we found that the expression of CD11b+CD33+ MDSCs was positively correlated with AFP and hepatic fibrosis in patients with HCC. The carbohydrate structure of AFP can produce specificity when hepatocarcinogenesis occurs, which is significantly different from that of benign liver disease [34]. The liver of patients with HCC is often accompanied by severe liver fibrosis, even cirrhosis. This may suggest that, as the number of CD11b+CD33+ MDSCs in the liver increases, the immunosuppressive ability of CD11b+CD33+ MDSCs cells is enhanced, which could accelerate the advance of HCC. And consistent with our results, Hetta et al. had demonstrated that the frequency of MDSCs was positively correlated with ALT, AFP, and HCV viral load and negatively correlated with CD8 T-cell frequency in subsets in Egyptian patients with hepatitis C virus-related hepatocellular carcinoma [35], not just the degree of liver fibrosis and inflammation. We also found that globulin and lymphocytes were significantly negatively correlated with the expression of hepatic MDSCs in CLD patients. We speculate that the increase in the expression of MDSCs inhibits the immune response to chronic inflammation, resulting in a decrease in globulin and lymphocytes in chronic liver disease. In AIH patients, the expression of hepatic MDSCs was positively correlated with AST, ALT, and AFP. A similar phenomenon has also been reported in other liver diseases [35–37]. Ye et al. reported that MDSCs were increased in murine autoimmune hepatitis models which had higher levels of ALT and AST [37]. These results suggest that an increase in the expression of MDSCs is due to the degree of inflammation of the liver.

Taken together, the CD11b+CD33+ MDSC level is correlated with the biochemical parameters of CLD patients, including the degree of liver fibrosis and enzymes related to liver injury, indicating that the level of MDSCs may be associated with disease progression. We had not observed a similar phenomenon in NAFLD, CHB, and PBC. However, recent work highlighted their significance in many other pathological conditions. One study showed that CD33+ CD11b+ CD14+ CD15- MDSCs were elevated in PBC [38]. Another study showed that MDSCs play crucial roles in the regulation of CHB [39]. The differences in findings could be due to the limitation in our sample selection. In addition, the strong correlation between MDSCs and AFP in HCC patients suggests that AFP may drive MDSCs to expand. Furthermore, the correlation between MDSCs and clinical parameters, such as ALT, AST, and globulin, supports that the inflammatory status may be an alternative mechanism for MDSC expansion in CLD patients. Detailed analyses of the association between MDSCs and inflammatory cytokines in CLD patients need to be conducted to provide convincing evidence.

We acknowledge the following strengths and limitations of our current study. We used immunofluorescence to evaluate the expression of MDSCs in patients with chronic liver disease under different pathological conditions and analyzed the connection with common clinical serum parameters. We designed a clinical study with multiple pathological perspectives, which provided the possibility for elucidating the underlying mechanism of MDSCs in chronic liver disease. However, this study only provides preliminary evidence of an association between MDSCs and inflammation or fibrosis in CLD. These data do not suggest causality. Finally, more observations related to MDSC recruitment and differentiation should be added, such as growth factors, cytokines, and chemokines. Therefore, further multicenter studies including a larger number of patients with CLD are needed to validate these findings.

In conclusion, we figured the clinical parameters that are associated with the expression of MDSCS in HCC and AIH patients and clarified the relationships between a feature of MDSCS and liver stages of hepatitis activity or liver fibrosis stage.

## Figures and Tables

**Table 1:** Baseline characteristics of different parameter degrees in CLD patients. HC: healthy control; CHB: chronic hepatitis B; NAFLD: nonalcoholic fatty liver disease; HCC: hepatocellular carcinoma; PBC: primary biliary cholangitis; AIH: autoimmune hepatitis; BMI: body mass index; ALT: alanine aminotransferase; AST: aspartate aminotransferase; Alb: albumin; GGT: gamma-glutamyl transferase; CHOL: cholesterol; TG: triglyceride; FPG: fasting plasma glucose; UA: uric acid; TB: total bilirubin; DB: direct bilirubin; AFP: alpha fetoprotein; WBC: white blood cells; N: neutrophils; L: lymphocytes; PLT: platelet; G: stages of hepatitis activity; S: liver fibrosis stage.

	HC	CHB	NAFLD	HCC	PBC	AIH
*n*	12	24	18	13	16	27
Gender (*n* (%))						
Male	5 (41.6%)	16 (66.7%)	11 (61.1%)	9 (69.2%)	0 (00.0%)	3 (11.1%)
Female	7 (58.4%)	8 (33.3%)	7 (38.9%)	4 (31.8%)	16 (100.0%)	24 (88.9%)
Age (*m* (IQR))	44.5 (33.25, 50.5)	28 (25, 34)	32 (24, 54)	60 (47.5, 65.5)	53 (46, 56.5)	50.5 (45.25, 57.5)
BMI (kg/m^2^,$\bar{x}\pm s$)	22.67 ± 2.23	21.02 ± 1.47	26.8 ± 2.43	23.78 ± 1.98	22.93 ± 3.43	23.13 ± 2.73
ALT (IU/L, *m* (IQR))	19 (16, 20.5)	210 (115, 317.5)	148 (88, 206)	27 (21.5, 38.5)	78 (34.5, 118.5)	103 (50.25, 271.5)
AST (IU/L, *m* (IQR))	18 (15, 21.75)	98 (76.5, 133)	74 (46, 79)	35 (20.5, 53.5)	60 (38, 88)	110 (44.75, 298.25)
GGT (IU/L, *m* (IQR))	13.5 (10.75, 20.75)	71 (29, 229.5)	74 (50, 155)	53 (16.5, 265)	128 (59.5, 528)	283.5 (83, 439.25)
CHOL (mmol/L, $\bar{x}\pm s$)	5.13 ± 1.19	5.56 ± 0.68	5.46 ± 1.17	5.76 ± 1.89	5.14 ± 0.83	5.05 ± 1.17
TG (mmol/L, $\bar{x}\pm s$)	1.16 ± 0.35	1.21 ± 0.48	1.68 ± 0.9	1.25 ± 0.41	1.31 ± 0.62	1.66 ± 0.79
FPG (mmol/L, $\bar{x}\pm s$)	5.63 ± 0.71	4.53 ± 0.45	5.42 ± 0.58	6.06 ± 0.46	5.1 ± 0.72	5.18 ± 0.52
UA (*μ*mol/L, $\bar{x}\pm s$)	319.5 ± 42.56	330.76 ± 113.57	496.04 ± 72.65	369.02 ± 106.38	259.4 ± 46.85	250.17 ± 83.29
TB (mg/dL, $\bar{x}\pm s$)	15.3 ± 6.99	20.16 ± 8.34	23.73 ± 8.05	14.26 ± 1.98	15.62 ± 4.79	30.67 ± 23.31
DB (mg/dL, *m* (IQR))	3.05 (2.18, 3.25)	6.4 (5.35,9.15)	4 (2.7, 5.4)	2.8 (1.9, 3.65)	1.9 (1.35, 7.4)	8.9 (2.8, 19.55)
AFP (ng/mL, *m* (IQR))	3.38 (1.43, 4.82)	8.11 (3.08, 11.04)	2.34 (1.89, 4.75)	79.33 (5.71, 253.3)	1.86 (1.71, 3.125)	3.85 (2.13, 6.44)
WBC ($\bar{x}\pm s$)	5.2 ± 1.18	5.72 ± 1.06	6.79 ± 1.2	5.5 ± 2.45	5.22 ± 1.53	4.1 ± 0.93
N ($\bar{x}\pm s$)	3.1 ± 0.94	2.9 ± 1.06	3.91 ± 1.1	3.66 ± 2.76	2.82 ± 0.93	2.03 ± 0.67
L (*m* (IQR))	1.75 (0.98, 2.38)	2.1 (2, 2.35)	2.5 (1.7, 2.8)	1.2 (0.6, 2.2)	1.6 (1.45, 2.25)	1.35 (1, 1.88)
PLT ($\bar{x}\pm s$)	195.98 ± 139.56	192.2 ± 52.03	232.14 ± 81.1	239.4 ± 90.22	226 ± 33.9	176.5 ± 69.12
Stages of hepatitis activity						
G ≤ 1	12	0	1	2	1	0
G = 2	0	9	15	9	7	4
G = 3	0	7	2	2	8	17
G = 4	0	8	0	0	0	6
Liver fibrosis stage						
S ≤ 1	12	12	9	2	4	2
S = 2	0	9	6	3	5	11
S = 3	0	2	2	5	4	9
S = 4	0	1	1	4	3	5

## Data Availability

The data used to support the findings of this study are available from the corresponding author upon request.
